# Evaluation of antibody level against *Fusobacterium nucleatum* in the serological diagnosis of colorectal cancer

**DOI:** 10.1038/srep33440

**Published:** 2016-09-28

**Authors:** Hai-Fang Wang, Lin-Fang Li, Song-He Guo, Qiu-Yao Zeng, Fen Ning, Wan-Li Liu, Ge Zhang

**Affiliations:** 1 Department of Microbial and Biochemical Pharmacy, School of Pharmaceutical Sciences, Sun Yat-sen University, Guangzhou, China; 2 Department of Clinical Laboratory Medicine, Sun Yat-sen University cancer center, Guangzhou, China; 3 Guangzhou Institute of Pediatrics, Department of Obstetrics, Guangzhou Women and Children’s Medical Center, Guangzhou Medical University, Guangzhou, China

## Abstract

*Fusobacterium nucleatum* (*F. nucleatum*, Fn) is associated with the colorectal cancer (CRC). Fn-infection could induce significant levels of serum Fn-specific antibodies in human and mice. The objective of this study was to identify Fn-infection that elicit a humoral response in patients with CRC and evaluate the diagnostic performance of serum anti-Fn antibodies. In this work, we showed the mean absorbance value of anti-Fn-IgA and -IgG in the CRC group were significantly higher than those in the benign colon disease group and healthy control group (*P* < 0.001). The sensitivity and specificity of ELISA for the detection of anti-Fn-IgA were 36.43% and 92.71% based on the optimal cut-off. The combination of anti-Fn-IgA and carcino-embryonic antigen (CEA) was better for diagnosing CRC (Sen: 53.10%, Spe: 96.41%; AUC = 0.848). Furthermore, combining anti-Fn-IgA with CEA and carbohydrate antigen 19-9 (CA19-9) (Sen: 40.00%, Spe: 94.22%; AUC = 0.743) had the better ability to classify CRC patients with stages I-II. These results suggested that Fn-infection elicited high level of serum anti-Fn antibodies in CRC patients, and serum anti-Fn-IgA level may be a potential diagnosing biomarker for CRC. Serum anti-Fn-IgA in combination with CEA and CA19-9 increases the sensitivity of detecting early CRC.

Commensal bacteria in the colon may play a role in colorectal cancer (CRC) development^1^. Accumulated studies show that several bacterial species seem to be involved in pathogenesis of CRC^2^. *Fusobacterium nucleatum* (*F. nucleatum,* Fn) is an opportunistic commensal anaerobe in the oral cavity which is also prevalent in human CRC tissues^3^, and is over-represented in disease tissue versus matched normal tissue in CRC patients^4^,^5^. Moreover, an overabundance of Fn has been reported to be associated with colorectal adenomas^6^, and show the potential as a risk factor for disease progression from adenoma to cancer^7^. Recently, multiple studies further identified the increased carriage of Fn in mucosal or fecal samples of CRC patients by 16 s RNA sequencing and quantitative PCR (qPCR) analysis^1^,^8^,^9^. The amount of Fn in CRC tissue has been proved to be associated with proximal tumor location and CpG island methylator phenotype (CIMP) status, microsatellite instability (MSI), and gene mutations in BRAF, KRAS and so forth^10^,^11^,^12^. Furthermore, a study has demonstrated that combining qPCR measurements of Fn with other several bacterial species had the ability to accurately diagnose patients with CRC^13^. In addition, it was demonstrated that Fn DNA was enriched in early-stage (I-II) patient and had the potential as a non-invasive early diagnostic biomarker for CRC from faecal samples^13^.

In general, microbe antigens can elicit a humoral immune response *in vivo.* In infection-associated cancers, a promising approach for the early detection of cancer is the assessment of immune response to antigens of tumor-associated microbe. Serological testing of antibodies against cancer-associated microorganisms including Epstein–Barr Virus (EBV), human papillomavirus (HPV) and *Helicobacter pylori* (Hp) has been used in diagnosis of the infection and tumor screening^14^,^15^. Previous studies reported that Fn induced significant humoral antibody response in human and mice with chronic oral Fn-infection^16^,^17^,^18^,^19^. The elevated antibodies level of Fn is also a risk factor for Alzheimer’s disease and rheumatoid arthritis^20^,^21^,^22^. Moreover, *F. nucleatum* immunization prior to that of *P. gingivalis* inhibited the production of anti-*P. gingivalis* antibodies, suggest that *F. nucleatum* do not allow the production of cross-reactive antibodies to other similar oral microorganisms^23^.

CRC incidence has increased at an alarming rate over the last twenty years^24^. Most cases of CRC are curable if diagnosed early enough, survival rates for early stage detection is about five times that of late stage cancers^25^. As a consequence, there is an urgent need to explore valuable early diagnosis markers for CRC patients. In the present study, we measured the preoperative anti-Fn levels in CRC patients to evaluate the clinical value of anti-Fn as a diagnostic parameter in those patients with colon cancer.

## Results

### Anti-Fn antibodies in sera of CRC patients with Fn infection

**Figure 1:**
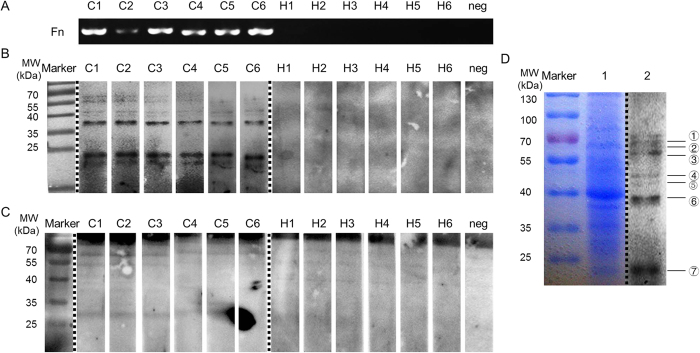
Detection of the specific antigen of Fn that leads to an intense immune response of CRC patients. (**A**) PCR was detected by PCR in stool of 6 CRC patients and healthy controls respectively (C: CRC patients; H: healthy controls; neg: negative control). (**B**,**C**) Antigens reactive with anti-Fn-IgA (**B**) and anti-Fn-IgG (**C**) were determined by western blotting through incubating with a reference serum dilution of 6 Fn-positive CRC patients or 6 Fn-negative healthy individual as primary antibody. (**D**) The whole proteins of Fn were separated by 10% SDS-PAGE and then stained with Coomassie brilliant blue (lane 1) and the specific antigens that caused high levels of anti-Fn-IgA were detected by western blotting through incubating with mixed serum samples of 6 CRC patients as primary antibody (lane 2). Of note, Fig. 1B–D were cropped from a single image on the dashed lines to be better presented in the article’s context. The gels have been run under the same conditions and subsequently processed with the same set of materials. These three complete figures could be found respectively in the Supplementary Figs S1 and S2.

To investigate the presence of antibodies against Fn in sera of CRC patients, we first screened Fn infection by PCR from the stool samples of 10 CRC patients and 10 matched healthy controls (Fig. 1A). 6 Fn-positive samples from CRC patients and 1 positive sample from healthy controls were detected. Sera of 6 Fn-positive patients and equal numbers of Fn-negative healthy controls were used to detect the specificity reactive antigens with the serum by western blotting. Several strong reactive antigen bands were observed with all Fn-positive sera samples when the serum was diluted 1:10000 and incubated with HRP-IgA, but no obvious bands were observed to react with sera of 6 healthy controls (Fig. 1B). However, it presented some nonspecific reactivity bands when followed with HRP-IgG (Fig. 1C). Additionally, to determine whether the serum antibodies were specific to Fn, we conducted the parallel experiments to detect the antibodies to the four control microorganisms. No obvious band was detected in *B. fragilis, E. coli*, *E. cloacae* or *E. Faecalis* with Fn positive or negative serum when diluted at 1:10000 and incubated with HRP-IgA (data not shown).

7 obviously reactive antigen bands in a molecular mass range from 15 kDa to 75 kDa were chosen as the interest proteins which triggered a strong anti-Fn-IgA response. Those corresponding proteins were extracted from the gels following SDS-PAGE and coomassie brilliant blue R250 staining (Fig. 1D). These proteins were digested with trypsin, and the resulting peptides were analyzed by MALDI-TOF/TOF analyzer. The corresponding spectra were used for a protein search in the NCBI and ExPASy databases. 7 major proteins identified by bioinformatics are shown in Table 1. The two corresponding proteins which present the strongest reactive bands are Alkyl hydrogen peroxide C (21.2 kDa) and major outer membrane protein FomA (42.3 kDa).

Those results suggested that Fn protein can elicit a strong humoral response and produce high levels of specific antibodies in CRC patients. The serum anti-Fn-IgA and anti- Fn-IgG showed an ability to diagnose the Fn infection.

### Evaluation of serum anti-Fn levels in patients with CRC

**Figure 2:**
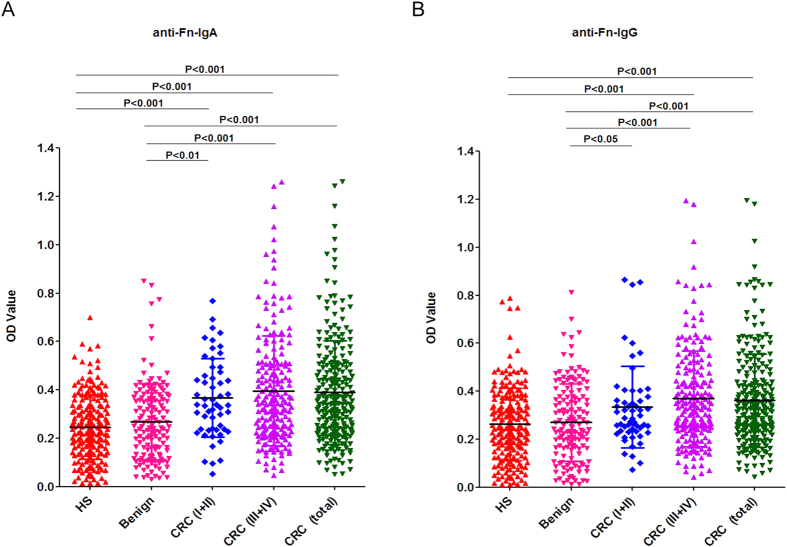
Comparison of OD values of anti-Fn-IgA or anti-Fn-IgG in sera from healthy adult human subjects (HS: healthy subjects, n = 200), benign colon disease (n = 150), stage I-II of CRC (n = 55), stage III-IV of CRC (n = 203), the total of CRC patients (n = 258) were individually assayed. Symbols indicate individual OD value; horizontal lines indicate mean values ± SD. Differences between the five groups were analyzed by Kruskal-Wallis test. (**A**) anti-Fn-IgA. (**B**) anti-Fn-IgG.

Furthermore, anti-Fn level was investigated in sera samples by indirect whole-cell ELISA. The coated bacteria whole cells served as an antigen to react with sera of patients and controls for detecting potential antibodies present in the sera. A total of 258 patients with CRC (55 in stage I-II) and two control groups (150 benign colon disease and 200 healthy controls) were recruited. CRC patients infected with Fn produce higher level of IgA than IgG. The average absorbance ± SD was observed using the 1:1000 diluted sera in IgA and 1:4000 diluted in IgG. As shown in Fig. 2A, the average absorbance ± SD for anti-Fn-IgA in CRC groups and its stage I-II of CRC groups was 0.390 ± 0.215, 0.367 ± 0.163, respectively; while in healthy controls and benign colon disease group was 0.246 ± 0.132, 0.268 ± 0.158 respectively. The circulating levels of anti-Fn-IgA in patients with CRC group and its stage I-II groups were significantly higher than those of the two control groups (*P* < 0.001), and there was no significant difference in the benign colon disease group and healthy control group. In addition, the circulating levels of anti-Fn-IgG in patients with CRC group (0.362 ± 0.194) were significantly higher than those of two control groups (0.262 ± 0.152; 0.270 ± 0.162), but there was no significant difference in stage I-II and control group (Fig. 2B). Although the highest levels were observed in later stage CRC (stage III-IV), the serum levels of anti-Fn-IgA and anti-Fn-IgG of CRC patients in stage I-II were not significantly difference than stage III-IV (*P* = 0.890; *P* = 0.200). To determine whether the serum antibodies were specific to Fn, we conducted the parallel-controlled assays by using whole cell ELISA. Fig. S3A–C showed that the OD value of anti-*B. fragilis,* anti-*E. coli* and anti-*E. cloacae*-IgA or -IgG were too low to be detected when applied diluted sera (1:1000 for anti-IgA or 1:4000 for anti-IgG level detection) from both patients and controls. Though the level of anti-*E. Faecalis*-IgA or -IgG were much higher than the other three bacterial strains, it showed no significant difference between CRC and two control groups (Fig. S3D).

### Association between antibodies against Fn and clinicopathological, serological characteristics of CRC patients

The data presented in Table 2 shows the relationship between anti-Fn and clinicopathological variables in CRC patients. The anti-Fn-IgA and anti-Fn-IgG were not obviously correlated with age, gender, tumor volume, T classification, N classification, metastasis and clinical stage. However, there was a significant association between the presence of anti-Fn-IgG and CRC histological differentiation (*P* = 0.032). The level of anti-Fn-IgG was higher in CRC patients with poorly differentiated adenoma than those with good differentiation. These results indicated that anti-Fn level is similar in CRC patients with advanced stage and in patients with early stage.

Furthermore, Pearson’s correlation coefficient and linear regression analysis were applied to analyze the correlation among the levels of anti-Fn-IgA, anti-Fn-IgG and the tumor markers CEA and CA19-9. Anti-Fn-IgA had a positively correlation with Anti-Fn-IgG (R = 0.365, *P* < 0.001). Neither CEA nor CA19-9 was associated with anti-Fn-IgA or -IgG levels (data not shown). In addition, anti-Fn-IgA and -IgG levels were similar in both CEA-positive and CEA-negative CRC patients, or in CA19-9-positive and CA19-9-neagtive CRC patients. These results suggested that anti-Fn-IgA and -IgG level were not associated with the progression of the CRC, but anti-Fn-IgA and -IgG level may provide an additional diagnostic value for those patients with CEA- negative or in early stage of CRC.

### Anti-Fn antibody levels have a diagnostic value for CRC

**Figure 3:**
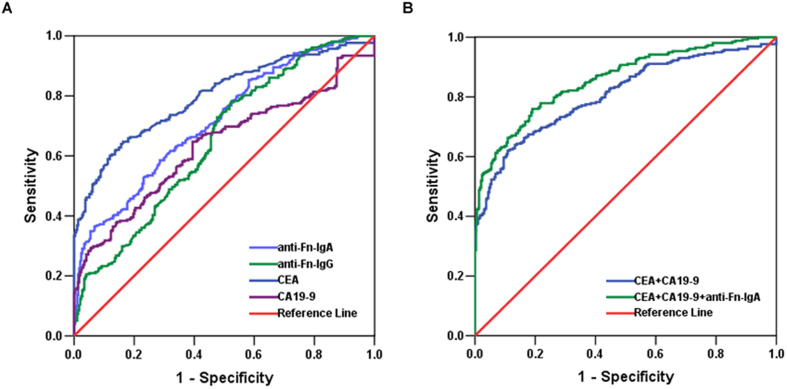
Diagnostic outcomes for serum anti-Fn-IgA, anti-Fn-IgG, CEA or CA19-9 alone or in combination in the diagnosis of CRC. (**A**) ROC curves for the diagnostic strength to identify CRC using anti-Fn-IgA, anti-Fn-IgG, CEA or CA19-9 level. (anti-Fn-IgA: AUC = 0.704; anti-Fn-IgG: AUC = 0.645; CEA: AUC = 0.796; CA19-9: AUC = 0.635). (**B**) ROC curves for the diagnostic strength to identify CRC using anti-Fn-IgA, CEA or CA19-9. (CEA+ CA19-9: AUC = 0.809; anti-Fn-IgA + CEA+ CA19-9: AUC = 0.858).

To determine whether levels of antibodies against Fn had diagnostic value for CRC, the ROC curve was plotted to identify a cut-off value that would distinguish CRC from both of nonmalignant colon diseases and health controls (Fig. 3; Table 3). As shown in Fig. 3, the area under the ROC curve (AUC) for anti-Fn-IgA and anti-Fn-IgG was 0.704 (95% CI: 0.663–0.746) and 0.645 (95% CI: 0.601–0.688), with an optimal cut-off value 0.420 and 0.230 respectively, whereas the AUCs for CEA and CA19-9 were 0.796 (95% CI: 0.758–0.834) and 0.635 (95% CI: 0.587–0.683), respectively. As shown in Table 3, the sensitivity of anti-Fn-IgA was 36.43%, which is higher than that for CA19-9 (20.16%), but lower than CEA (41.47%), whereas the specificity of anti-Fn-IgA was slightly lower (92.71%) based on the optimal cut-off (0.42) according to the Youden Index. The sensitivity of anti-Fn-IgA (30.62%) is reduced with the further adjustment of cut-off value (0.45) based on the similar specificity with CEA (about 96%) in this study. Anti-Fn-IgG demonstrated lower sensitivity and specificity, similar to that of CA19-9. Moreover, Table 3 demonstrated that the sensitivity of the combination of anti-Fn-IgA and CEA was superior to that of the combination of CEA and CA19-9 (53.10% *vs*. 45.74%) without compromising the specificity (96.41% *vs.* 96.26%) by using a higher adjustment cut-off score (0.70). In addition, the combination of anti-Fn-IgA and CEA exhibited a similar PPV (positive predictive value, 92.6% *vs*. 91.5%) and higher NPV (negative predictive value, 70.9% *vs.* 66.9%) compared with the combination of CEA and CA19-9. The combination of anti-Fn-IgA with CEA and CA19-9 slightly increased the diagnosis value compared with the combination of anti-Fn-IgA and CEA. These results suggest that IgA, but not IgG against Fn possess diagnostic capabilities for CRC, and anti-Fn-IgA increases the sensitivity in CRC detection and offers additional diagnosis value in CRC.

### Diagnostic value of individual serum anti-Fn, CEA and/or CA19-9 levels in the detection of early stage CRC

The level of anti-Fn-IgA was similar in CRC patients with advanced stage and early disease. Due to the lack of biological markers for detecting early stage colorectal cancer, to investigate the early diagnostic value of individual serum anti-Fn is valuable.

**Figure 4:**
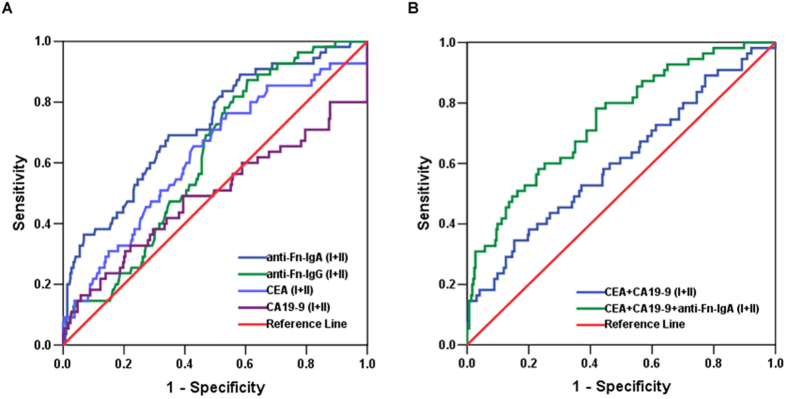
Diagnostic outcomes for serum anti-Fn-IgA, anti-Fn-IgG, CEA or CA19-9 alone or in combination in the diagnosis of early stage CRC. (**A**) ROC curves for the diagnostic strength to identify early CRC using anti-Fn-IgA, anti-Fn-IgG, CEA or CA19-9 level. (anti-Fn-IgA: AUC = 0.709 anti-Fn-IgG: AUC = 0.612; CEA: AUC = 0.624; CA19-9: AUC = 0.492). (**B**) ROC curves for the diagnostic strength to identify CRC using anti-Fn-IgA, CEA or CA19-9. (CEA+ CA19-9: AUC = 0.605; anti-Fn-IgA + CEA+ CA19-9: AUC = 0.743).

The performance of anti-Fn and CEA or CA19-9 in detecting early stages (stage I, stage II) of CRC was performed in 55 patients with CRC at early stages. As shown in Fig. 4, the AUC of anti-Fn-IgA and anti-Fn-IgG reached 0.709 (95% CI: 0.635–0.784), and 0.612 (95% CI: 0.543–0.681), respectively, whereas the AUCs for CEA and CA19-9 were only 0.624 and 0.492. Table 4 demonstrated the sensitivity and specificity of anti-Fn-IgA was 69.09% and 63.27% based on the optimal cut-off (0.28), and the sensitivity was 27.27% with the higher adjustment cut-off score (0.45) without compromising the specificity (96.21%), but CEA and CA19-9 were detected in 9 patients and the sensitivity was only 16.36% (AUC = 0.605). By combining with the anti-Fn-IgA, CEA and CA19-9, the sensitivity and specificity reached 40.0% and 94.22% based on the optimal cut-off (AUC = 0.743), and exhibited a higher PPV (56.4% *vs*. 29.7%) and NPV (89.4% *vs.* 87.4%) than combining CEA with CA19-9 alone. The findings validate the performance of anti-Fn-IgA as a plasma marker to increase the diagnostic sensitivity for early detection of CRC, and indicate that the serum levels of anti-Fn-IgA, CEA and CA19-9 combined have a better diagnosis values for screening early stage of CRC than CEA and CA19-9 combined diagnosis.

## Discussion

In this study, we demonstrated higher levels of polyclonal antibodies against Fn present in the blood of CRC patients with Fn-infections compared to healthy controls. This finding indicated that Fn-infection induced a specific and stronger humoral antibody in patients with CRC.

In mice, vaccination of heat-killed whole bacteria sonic extracts with Fn induced a specific humoral antibody response and produced specific higher titer antibodies than other oral bacteria^26^. Recently, the FomA porin from Fn was identified as a toll-like receptor 2 (TLR2) agonist with immune adjuvant activity. FomA induces TLR2-dependent antigen-specific antibody secretion by mouse B cell activation *in vivo*^27^, which was in line with our findings that Fn-infection patients drive stronger immune responses than healthy controls.

Antibody levels to Fn were also found to be significantly increased in patients with Alzheimer’s disease compared with controls by whole Fn cell ELISA assay^22^. The commensal intestinal microbes play a crucial role in the bidirectional gut-brain axis that integrates gut and central nervous system activities. A dysfunction of this axis has been associated with the pathogenesis of peripheral and central nervous system diseases^28^,^29^. The result further shows that digestive tract infection induced the higher titer of Fn antibodies in humans.

Fn has historically been viewed as an oral pathogen, multiple studies have shown that the antibody levels of whole Fn cell is able to diagnose Fn-infection oral patients. Although it could be suggested that the antibody to these pathogens may have been cross-reactive with antigens from other sources, the previously published data are replete with oral pathogen studies supporting the specificity of these antibodies for oral infections^30^,^31^,^32^,^33^,^34^, and that successful treatment and maintenance of periodontitis significantly lowers these antibody levels^35^. In addition, *H. pylori* Whole-Cell antigens ELISA was used to detect the anti-Hp level previously and the results do not show much difference with its anti-CagA ELISA in multiple studies^36^,^37^.

Specific antibodies have been widely used to diagnosis infection. Some cancer-associated microorganisms, which enter through mucosal infection sites, present specific IgA antibodies for diagnosis and screening. CagA antibodies were significantly more prevalent among individuals with elevated H. pylori antibody titers of the IgA class than in those with IgG antibodies^38^. EBV-IgA related and HPV-IgA related serological testing has been used for nasopharyngeal carcinoma and cervical carcinoma diagnosis^39^,^40^. Our study also shows that IgA has higher titer than IgG, suggesting the IgA class may be more valuable for diagnosis CRC than the IgG class.

Furthermore, we demonstrated significantly higher anti-Fn-IgA and anti-Fn-IgG levels were present in the serum of CRC patients with Fn infection compare to the healthy controls and or patients with benign colon disease. Recently, numerous studies have indicated that Fn is closely associated with the development and progression of CRC^4^,^5^,^6^,^7^. Our data were consistent with those studies in which Fn-DNA is overabundant in CRC patients^1^,^8^,^9^. Moreover, we showed that anti-Fn-IgA and anti-Fn-IgG were significantly elevated in early stage CRC compared with normal controls or those with benign disease, but only slightly elevated when compared early stage CRC to advanced CRC. These data are in line with the study in which Fn-DNA was enriched in early-stage CRC patients^13^, corresponding to a greater lag time in the production of antibodies in response to infection.

Early diagnosis and treatment of CRC is of great value to improve survival of CRC patients. Currently, CEA and CA19-9 are the two most commonly used diagnosis markers for CRC. However, serum tests such as CEA or CA19-9 have poor sensitivity for detection of early CRC. In our study, Fn-IgA showed higher sensitivity (36.43%) than CA19-9 (20.16%), and anti-Fn-IgA combined with CEA and CA19-9 improved the sensitivity (54.65%) without compromising specificity (96.60%) for CRC detection. These results show the diagnostic value of CRC with anti-Fn-IgA. Furthermore, anti-Fn-IgA exhibited much higher sensitivity (27.27%) than CEA (9.09%) or a combination of CEA and CA19-9 (16.36%) for early CRC detection without compromising specificity (96.21%). The combination of anti-Fn-IgA, CEA and CA19-9 exhibited highest sensitivity (40.0%) and specificity (94.22%) for early CRC detection with highest PPV (56.4%) and good NPV (89.4%). These results demonstrated that the combination of anti-Fn-IgA, CEA and CA19-9 could detect about 40% early CRC, and the improvement in sensitivity compared to CEA was accomplished without obviously compromising specificity. Therefore, anti-Fn-IgA is a valuable serological diagnosis marker for screening early CRC.

Fn DNA load quantified from stool is inconvenient as a large scale population screening assay. Serologic testing for Fn antibodies will be a simpler diagnosis method and may be useful to monitor population exposure to Fn. The identification of microbial antigens that elicit an immune response is important for clinical applications, and microbial antigens may be used for early diagnosis, prognosis, and immunotherapy against the disease with cancer-associated microorganisms. The development of antibody detection for Fn is valuable for prospective epidemiological surveillance and large-scale screening of early CRC. In recent years, mSeptin 9 blood test has been a promising biomarker for detection of colorectal cancer due to its high sensitivity (about 70%), high specificity (above 90%) and its cost-effectiveness^41^,^42^,^43^. To improve the sensitivity and specificity for the diagnosis of early CRC, the combination of single or multivalence Fn antigens with mSeptin 9 is worthy of further study.

In summary, Fn infection can elicit a strong humoral immune response. The levels of anti-Fn-IgA and anti-Fn-IgG in sera from CRC patients were significantly higher than those from healthy subjects and benign colon disease. Moreover, our study demonstrated that serum levels of anti-Fn-IgA combined with CEA and CA19-9 have a superior sensitivity than CEA and CA19-9 combined alone in detecting early stage of CRC. Therefore, Fn-IgA antibodies become a potential and useful screen marker for early CRC.

## Materials and Methods

### Patients, blood and stool samples

Sera were collected between June 2013 and December 2013 from 258 patients with primary CRC at the time of diagnosis before tumor resection at the Cancer Center of Sun Yat-sen University. Diagnosed CRC patients had been histological confirmed by biopsy and further tests, including stool examination, X-rays, endoscope examination and magnetic resonance imaging (MRI). The CRC cohort consisted of 144 male patients and 114 female patients. Patients ranged in age from 12 to 94 years (mean, 54.8 years). The stages of disease progression were classified according to the 2009 Union for International Cancer Control (UICC) classification. The CRC group included 34 patients with stage I, 21 with stage II, 109 with stage III, and 94 with stage IV. The patient characteristics were described in Table 2.

Serum of 150 patients (ages 16–82 years, median = 56.8 years, 87 males and 69 females) with benign colon disease (142 cases of adenomatous polyps, 8 cases of ulcerative colitis) were collected at the first affiliated hospital of Sun Yat-sen University. Benign colon disease was diagnosed based on standard endoscopic, histologic, and radiographic criteria.

Sera from 200 healthy volunteers without inflammation (112 males, 88 females) with ages ranging from 18 to 74 years (mean: 54.0 years) were collected from the physical examination department at the Cancer Center of Sun Yat-Sen University and used as controls. Healthy controls and benign colon disease were selected from an archive of blood samples; the control samples were matched as closely as possible to the CRC group for sex, previous handling and the time period of sample collection.

The stool and corresponding sera samples which were investigated for specificity of the antibodies against Fn and *B. fragilis*, *E. coli, E. cloacae and E. Faecalis* proteins, were collected from 10 CRC patients and 10 health volunteers at the Cancer Centre of Sun Yat-sen University in June 2013.

A 5-ml blood sample from each participant was allowed to clot for 30 to 60 min at room temperature. Each clotted sample was centrifuged at 4000 rpm for 10 min. All sera were then aliquoted and frozen at −80 °C until use.

### Compliance with ethical standards

Ethics approvals were granted by the Ethics Committee of Sun Yat-sen University Cancer Center (No. GZR2012-123) and the Ethics Committee of the first affiliated hospital of Sun Yat-sen University (No. [2012]396), with all methods carried out in accordance with the approved guidelines. Written informed consent was required for all patients enrolment into the study.

### Bacterial Cultures

*F. nucleatum* strain ATCC 25586, *B. fragilis* strain ATCC 25285, *E. coli* strain ATCC 25922, *E. cloacae* strain ATCC 700323 and *E. Faecalis* strain ATCC 29212 were purchased from Institute of Microbiology of Chinese Academy of Sciences. Fn 25586 and *B. fragilis* 25285 were grown anaerobicly at 37 °C for 72 h and 48 h respectively in CDC anaerobic blood agar plate (Guangzhou detgerm Microbiology Technology Co.LTD, China) or 48 h in brain heart infusion (Oxoid, UK) broth culture before harvesting, while *E. coli*, *E. cloacae* and *E. Faecalis* strains were cultured aerobically at 37 °C for 24 h in LB agar plate.

### PCR

DNA from stool samples of CRC patients and health controls was extracted by E.Z.N.A stool DNA kit (Omega Bio-Tek, USA) according to the manufacture’s instructions. PCR for Fn genes was performed on a PCR Amplifier (BIO-RAD C1000, USA). The primers used in real-time PCR reaction were as follows: forward: 5′-ATA CCG GGA ATA AAG ACA-3′; reverse: 5′-TAC AAC CCA ATC CAT AAG T -3′.

### Western Blot

*F. nucleatum* were collected, resuspended in 0.01 M PBS and disrupted by hypothermic ultrasonication, then total protein was extracted. Equivalent protein amounts were denatured in a 2 × SDS loading buffer, and separated by 10% SDS-PAGE, followed by transferring onto polyvinylidene difluoride (PVDF) membrane. *B. fragilis, E. coli, E. Faecalis, and E. cloacae* were under the same process and used as controls. After being blocked with 5% non-fat dry milk in PBS containing 0.05% Tween-20, the membrane strips were incubated with serum from Fn-positive CRC patient or Fn-negative healthy individual (1:10000) at 4 °C overnight. After washing for several times, the PVDF membrane strips were incubated in horseradish peroxidase (HRP) labeled anti-human-IgA (1:20000, Boster, China) or -IgG (1:10000, Earthox, USA) for 2 h at room temperatures. The bands were detected by Pierce ECL Plus Western Blotting Substrate (Thermo Scientific, USA) according to the manufacturer’s suggested protocols.

### Antigen Identification

For antigen identification, the proteins of interest were excised from the gels and digested with trypsin (Promega, USA) by Mass Standards Kit for the 4700 Proteomics Analyzer (Applied Biosystems), then the tryptic protein hydrolysates were analyzed using 4800 Plus MALDI TOF/TOF^TM^ Analyzer (Applied Biosystems, USA). Protein identification was repeated at least twice using bands from two different gels. The obtained peptide mass fingerprint (PMF) was used by Mascot 2.2 software to search Swiss-Prot and NCBI nr protein databases.

### ELISA

Serum specific anti-Fn-IgA and -IgG level was determined by an indirect whole-cell ELISA. Briefly, 96-well microtitre plates were aldehyded with 2.5% glutaric dialdehyde at 37 °C for 2 h. Plates were washed with PBST for three times. Then, 100 μl heated-inactivated Fn at a final concentration of 1 × 10^8^ CFU/ml in 0.05 M Na_2_CO_3_-NaHCO_3_ buffer was added to each well. Then ELISA plates were incubated at 37 °C until the solution was dry. After that, each well was blocked with 200 μl of 1% BSA in PBST at room temperature for 2 h. For detection of anti-Fn-IgA level, 100 μl serum diluted at 1:1000 in block buffer were added in duplicates for 1 h at 37 °C. PBST washing for three times, then 100 μl goat anti-human IgA conjugated with HRP (Boster Biotechnology, China) diluted at 1:10000 was incubated for 30 min at 37 °C. For detection of anti-Fn-IgG level, 100 μl serum diluted at 1:4000 in block buffer were added in duplicates for 1 h at 37 °C. While 100 μl goat anti-human IgG conjugated with HRP (EarthOx, USA) was diluted at 1:10000 for incubation at 37 °C for 30 min. Wells of only block buffer were used for determination of background values. Finally, the substrate (tetramethylbenzidine) solution was added, and the reaction was terminated using 2 M H_2_SO_4_ and read at an OD of 450 nm by ELISA spectrophotometer (Bio-Rad, USA). The reproducibility of tests within-run and between-run was determined using the same pooled (n = 10) serum from CRC patients, which showed a strong immune response with Fn. The repetition CV% was less than 12%. All the OD value were required between 0.0–2.0 to adjust the optimal linear range. Parallel assays using *B. fragilis, E. coli, E. cloacae, and E. Faecalis* coating the ELISA plates were kept as controls to determine the specificity of the titers.

### CEA and CA19-9 assay

The concentrations of carcinoembryonic antigen (CEA) and carbohydrate antigen 19-9 (CA19-9) in the serum were assessed using electrochemiluminescence immunoassay (ECLIA) kits on a Roche E170 fully automatic electrochemistry luminescence immunity analyzer (Roche, Germany). Each test included a standard control (CV < 5%). The cut-off levels were 5.0 ng/ml and 35.0 U/ml for CEA and CA19-9, as recommended by the manufacturer.

### Statistical analysis

All statistical analyses were carried out using the SPSS 16.0 statistical software package (SPSS Inc., Chicago, IL). The relationships between the specific anti-Fn-IgG or -IgA antibodies and the clinicopathologic features were analyzed by the Mann-Whitney U test. And the comparisons of specific anti-Fn-IgG or -IgA antibodies among different groups were assessed using the Kruskal-Wallis test. The efficacy of specific anti-Fn-IgG or -IgA antibodies for diagnosis was evaluated by the area under receiver operating characteristic (ROC) curve (AUC). Cut-off value for anti-Fn-IgG or -IgA antibodies was defined as the value with the maximization of the Yuden index. Furthermore, sensitivity (Sen), specificity (Spe), positive predictive value (PPV) and negative predictive value (NPV) were used to compare the efficiency of diagnosis among anti-Fn-IgG or -IgA antibodies, CEA and CA19-9. All statistical tests were two-sided, and *P* < 0.05 was considered statistically significant.

## Additional Information

**How to cite this article**: Wang, H.-F. *et al*. Evaluation of antibody level against *Fusobacterium nucleatum* in the serological diagnosis of colorectal cancer. *Sci. Rep.* **6**, 33440; doi: 10.1038/srep33440 (2016).

## Supplementary Material

Supplementary Information

## Figures and Tables

**Table 1:** Proteins identified by MS and bioinformatics.

Lane NO.	Protein information		Accession No.a)	Protein MWb)	Protein PIc)	Pep. Countd)	Protein Scoree)	Protein Score C. I.%f)	Total Ion Scoreg)	Total Ion C. I.%h)	Sequence coverage (%)i)
	Rank	Protein Name									
①	1	Protein Translation Elongation Factor G (EF-G)	gi|19712779	77482.3	5.12	9	154	100	127	100	17
	2	Outer membrane protein	gi|19713165	76182.0	8.57	10	218	100	173	100	21
	3	Chaperone protein dnaK	gi|19713544	65456.8	4.96	7	106	100	75	100	20
②	1	Tryptophanase	gi|19713205	62451.6	5.52	12	244	100	174	100	36
③	1	(S)-2-hydroxy-acid oxidase chain D	gi|19712753	52475.7	4.85	4	147	100	133	100	8
	2	Dipeptide-binding protein	gi|19713870	57187.3	5.07	10	140	100	91	100	19
④	1	NAD-specific glutamate dehydrogenase	gi|19713973	48497.8	6.44	10	501	100	445	100	34
⑤	1	Methionine gamma-lyase	gi|19715077	43449.5	6.15	13	260	100	163	100	20
	2	acyl-CoA dehydrogenase	gi|19714324	41898.5	8.2	7	116	100	85	100	27
	3	Acetyl-CoA acetyltransferase	gi|19713982	42534.3	6.62	8	109	100	64	99.996	32
⑥	1	porin	gi|530296	42346.3	9.37	13	260	100	163	100	39
	2	Major outer membrane protein	gi|19713103	42347.2	9.24	13	260	100	163	100	36
⑦	1	Alkyl hydroperoxide reductase C22 protein	gi|19713242	21196.8	5.22	8	567	100	500	100	48
	2	SSU ribosomal protein S4P	gi|19714919	22408.2	10.06	4	99	100	74	100	29

^a)^Protein accession number on NCBI.

^b)^Protein molecular weight.

^c)^Protein isoelectric point.

^d)^The number of matching peptides.

^e)^The score of protein.

^f)^Protein credibility.

^g)^The total ion score.

^h)^The total ion credibility.

^i)^The number of indentified amino acids against the total number of amino acids in the target protein.

**Table 2:** Relationship between the OD value of IgA/IgG antibodies against *F. nucleatum* and the clinicopathological variables in 258 patients with colorectal cancer.

	n	IgA (Mean ± SD)	P	IgG (Mean ± SD)	P
Gender					
Male	144	0.381 ± 0.215	0.434	0.344 ± 0.189	0.065
Female	114	0.400 ± 0.217		0.384 ± 0.200	
Age (y)					
<60	153	0.383 ± 0.213	0.401	0.366 ± 0.208	1.000
≥60	105	0.401 ± 0.220		0.355 ± 0.172	
tumor volume (cm3)					
≥8.0	83	0.425 ± 0.248	0.070	0.366 ± 0.190	0.816
<8.0	175	0.373 ± 0.197		0.360 ± 0.197	
Histological differentiation					
Well	25	0.401 ± 0.239	0.658	0.288 ± 0.157	0.032
Moderate	171	0.373 ± 0.188		0.350 ± 0.172	
Poor	62	0.431 ± 0.269		0.424 ± 0.244	
pT classification					
T1 + T2	65	0.373 ± 0.193	0.659	0.327 ± 0.157	0.102
T3 + T4	193	0.395 ± 0.222		0.373 ± 0.204	
pN classification					
No	152	0.393 ± 0.215	0.742	0.362 ± 0.180	0.567
Yes	106	0.385 ± 0.217		0.362 ± 0.214	
pMetastasis					
No	205	0.383 ± 0.211	0.351	0.356 ± 0.184	0.622
Yes	53	0.416 ± 0.230		0.383 ± 0.231	
Stage					
I + II	55	0.367 ± 0.163	0.890	0.333 ± 0.170	0.200
III + IV	203	0.396 ± 0.227		0.369 ± 0.200	
CEA (μg/ml)					
<5	146	0.381 ± 0.225	0.156	0.356 ± 0.183	0.786
≥5	112	0.400 ± 0.203		0.369 ± 0.209	
CA19-9 (U/ml)					
<35	206	0.394 ± 0.220	0.638	0.368 ± 0.197	0.407
≥35	52	0.371 ± 0.195		0.337 ± 0.182	

**Table 3:** The diagnosis value of CEA, CA19-9 and antibodies against *F. nucleatum* in CRC.

Combinations	Criterion	Sensitivity (%)	Specificity (%)	PPV (%)	NPV (%)
CEA	5.07	41.47	96.41	90.7	66.1
CA19-9	35.12	20.16	98.30	91.2	58.4
anti-Fn-IgA	0.42	36.43	92.71	79.0	66.0
	0.45	30.62	96.21	85.9	64.8
anti-Fn-IgG	0.23	77.52	46.94	52.4	73.5
	0.51	18.60	96.21	78.7	61.1
CEA+CA19-9	0.47	62.02	89.12	83.3	72.8
	0.72	45.74	96.26	91.5	66.9
anti-Fn-IgA+CEA	0.45	69.38	83.99	78.5	76.5
	0.70	53.10	96.41	92.6	70.9
anti-Fn-IgA+CEA+CA19-9	0.39	75.97	80.61	77.5	79.3
	0.72	54.65	96.60	93.7	71.0

**Table 4:** Diagnostic values of CEA, CA19-9 and antibodies against *F. nucleatum* in early stage CRC.

Combinations	Criterion	Sensitivity (%)	Specificity (%)	PPV (%)	NPV (%)
CEA	5.05	9.09	96.41	31.3	85.5
CA19-9	35.12	7.3	98.30	32.3	90.5
anti-Fn-IgA	0.28	69.09	63.27	23.2	92.7
	0.45	27.27	96.21	53.6	89.2
anti-Fn-IgG	0.20	87.27	37.03	18.2	94.8
	0.51	12.73	96.21	35.0	87.3
CEA+CA19-9	0.18	34.55	84.69	29.7	87.4
	0.28	16.36	96.43	46.5	86.1
anti-Fn-IgA+CEA	0.15	62.27	67.97	27.4	92.0
	0.31	27.27	96.40	58.1	88.1
CEA+CA19-9 + anti-Fn-IgA	0.29	40.0	94.22	56.4	89.4
	0.32	27.27	96.60	60.4	87.7
