# Supplementary material for: Evaluation of antibody level against Fusobacterium nucleatum in the serological diagnosis of colorectal cancer
Source: Sci Rep. 2016 Sep 28;6:33440. doi: 10.1038/srep33440 (PMC5039407; doi:10.1038/srep33440)
Supplement: Supplementary Information [file srep33440-s1.pdf]

# Evaluation of antibody level against *Fusobacterium nucleatum* in the serological diagnosis of colorectal cancer

Hai-Fang Wang[1#], Lin-Fang Li[2#], Song-He Guo[1], Qiu-Yao Zeng[2], Fen Ning[3], Wan-Li Liu[2], Ge Zhang[1*]

[1]Department of Microbial and Biochemical Pharmacy, School of Pharmaceutical Sciences, Sun Yat-sen University, Guangzhou, China

[2]Department of Clinical Laboratory Medicine, Sun Yat-sen University cancer center, Guangzhou, China

[3]Guangzhou Institute of Pediatrics，Department of Obstetrics, Guangzhou Women and Children's Medical Center，Guangzhou Medical University，Guangzhou, China

[#] Hai-Fang Wang and Lin-Fang Li contributed equally to this work.

[*] **Corresponding Author:** Ge Zhang, Department of Microbial and Biochemical Pharmacy, School of Pharmaceutical Sciences, Sun Yat-sen University, No.132 Waihuandong Road, University Town, Guangzhou 510006, China. Tel: 86-20-39943027; Fax: 86-20-39943021; E-mail: zhangge@mail.sysu.edu.cn

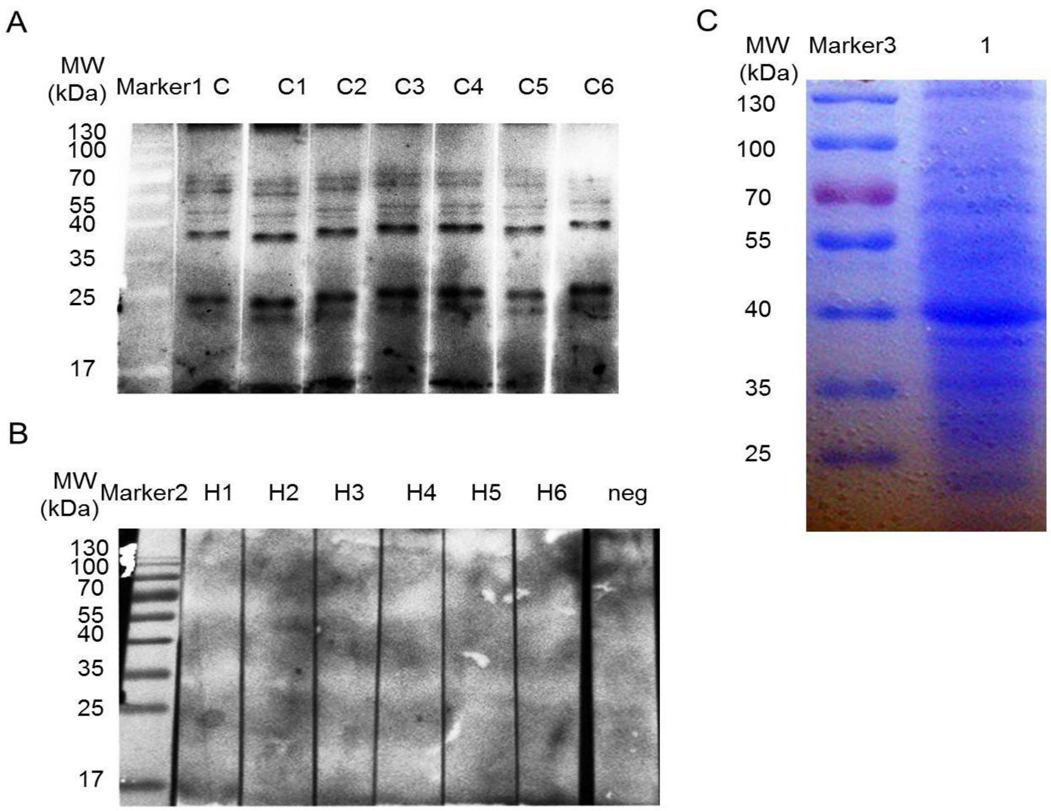


**Figure S1:** Complete figures of the antigens reactive with anti-Fn-IgA in Fig. 1B and Fig. 1D were shown. (A) The membrane strips were incubated with pooled serum (lane C) or separated serum from Fn-positive CRC patients (lane C1-C6); (B) The membrane strips were incubated with Fn-negative healthy individual (lane H1-H6) or blocking reagent as negative control (lane neg); (C) The whole proteins of Fn were separated by 10% SDS-PAGE and then stained with Coomassie brilliant blue (lane 1). The gels in Fig. S1 have been run under the same conditions and the Western blot performed with the same set of materials. Fig. S1B and lanes C1-C6 in Fig. S1A were included in Fig. 1B of this manuscript; Fig. S1C and lane C in Fig. S1A were included in Fig. 1D of this manuscript.

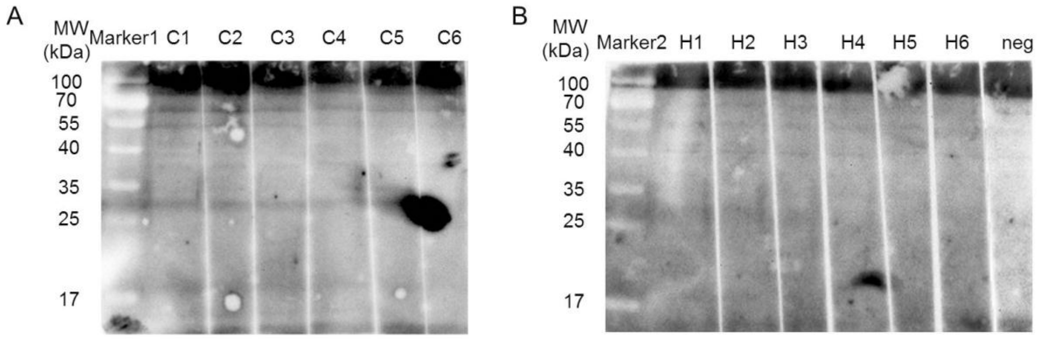


**Figure S2:** Complete figure of the antigens reactive with anti-Fn-IgG in Fig.1C was shown. (A) The membrane strips were incubated with separated serum from Fn-positive CRC patients (lane C1-C6); (B) The membrane strips were incubated with Fn-negative healthy individual (lane H1-H6) or blocking reagent as negative control (lane neg). The gels in Fig. S2 have been run under the same conditions and the Western blot performed with the same set of materials. Fig. S2A, lanes H1-H6 and lane neg in Fig. S2B were included in Fig. 1C of this manuscript.

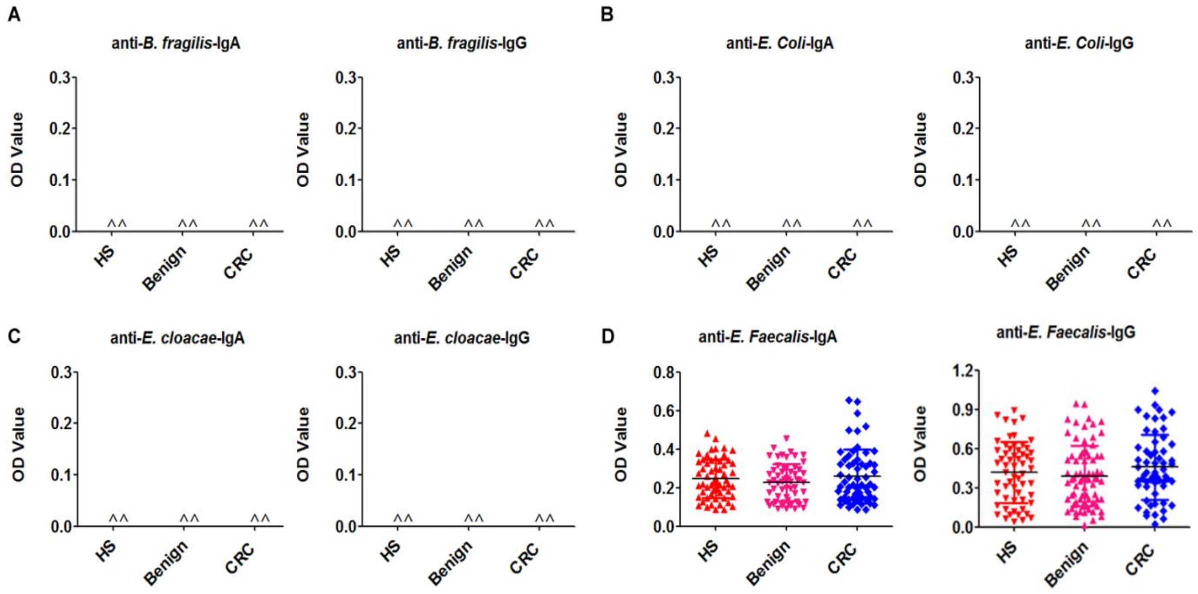


**Figure S3:** Comparison of OD values of anti-IgA or anti-IgG in sera from healthy adult human subjects (HS: healthy subjects, n = 60), benign colon disease (n = 60), CRC patients (n = 60) were individually assayed. Symbols indicate individual OD value; horizontal lines indicate mean values ± SD. Differences between the three groups were analyzed by Kruskal-Wallis test. (A) anti-*B. fragilis*-IgA or -IgG. (B) anti-*E. Coli*-IgA or -IgG. (C) anti-*E. cloacae*-IgA or -IgG. (D) anti-*E. Faecalis*-IgA or -IgG. “^^” means the OD value is below the detection limit.
